# Biliopancreatic diversion with duodenal switch improve polycystic ovary syndrome with decreased serum ceramides

**DOI:** 10.3389/fendo.2026.1720242

**Published:** 2026-02-05

**Authors:** Xiaochuan Li, Haomin Chen, Pengyu Li, Mingfei Wang, Dalin Wu, Juan Liu, Weijie Chen

**Affiliations:** 1 National Clinical Research Center for Women's Health and Obstetric and Gynecologic Diseases, Department of Obstetrics and Gynecology, Peking Union Medical College Hospital, Chinese Academy of Medical Sciences & Peking Union Medical College, Beijing, China; 2 Department of General Surgery, Peking Union Medical College Hospital, Chinese Academy of Medical Sciences, Beijing, China; 3 Shenzhen Campus of Sun Yat-Sen University, School of Bio Medical Engineering, Shenzhen, Guangdong, China; 4 Shenzhen Campus of Sun Yat-Sen University, School of Pharmaceutical Sciences (Shenzhen), Shenzhen, Guangdong, China

**Keywords:** bile acids, ceramides, glucose, polycystic ovary syndrome, surgery

## Abstract

**Background:**

Polycystic ovary syndrome (PCOS) is characterized primarily by insulin resistance and reproductive disorders. Biliopancreatic diversion with duodenal switch (BPD/DS) could significantly improve insulin sensitivity with significantly elevated level of bile acids. We aimed to explore changes in PCOS manifestations after BPD/DS and the possible mechanisms.

**Methods:**

Twenty PCOS rat models were assigned into the BPD/DS group and the sham BD (S-BPD) group randomly. The fasting blood glucose, bile acids, and ceramides levels in serum samples were assessed.

**Results:**

The reproductive disorders of BPD/DS group were almost completely restored 8 weeks after surgery, and the AUC_OGTT_ value and the AUC_ITT_ value were statistically less than those of the S-BPD group (*P* =0.001). The concentration of serum testosterone, luteinizing hormone, and follicle-stimulating hormone of the BPD/DS group was statistically less than that of the S-BPD group (*P <*0.05). Moreover, high level of bile acids and low level of ceramides were observed in the BPD/DS group (*P <*0.001). Bile acid sequestrant was given to the BPD/DS group rats for 1 week, the level of bile acids decreased and ceramides increased, the insulin resistance worsened. The AUC_OGTT_ value increased from 818.3 ± 297.3 mmol/L·min to 1147.9 ± 167.9 mmol/L·min (*P* =0.007) and the AUC_ITT_ value increased from 525.6 ± 52.3 mmol/L·min to 577.7 ± 102.9 mmol/L·min (*P* =0.023).

**Conclusions:**

Our study showed the improvement of insulin resistance in PCOS models after BPD/DS with decreased serum ceramides. The sequestrant of bile acid reversed the improvement of insulin resistance with higher ceramides level.

## 1 Introduction

Polycystic ovary syndrome (PCOS) is a kind of common endocrine and metabolic disorders in women, with the prevalence of 9 - 18% (1). Affected patients suffer a 15-fold increased risk of infertility and pregnancy complications including miscarriage, preeclampsia and preterm delivery (2). The characteristics of PCOS is insulin resistance and compensatory hyperinsulinism, which enhance androgen production and disturb menstrual cycle (3). Moreover, an elevated risk of obesity and type 2 diabetes (T2DM) was observed in PCOS patients. Ranging from 40 to 85% PCOS patients are either overweight or obese (4). Overweight in PCOS patients amplifies adverse metabolic outcomes with a 4-fold increased risk of T2DM (5).

Biliopancreatic diversion with duodenal switch (BPD/DS) is an efficacious operations for the control of obesity and T2DM (6). It could achieve 39.4% in total weight loss over 3 years compared with 29.6% for Roux-en-Y gastric bypass and 22% for sleeve gastrectomy (7). And T2DM patients maintain adequate glycemic control with fewer diabetes-related complications for 10 years after surgery (8). BPD/DS could significantly improve insulin sensitivity with significantly elevated level of bile acids (9, 10). BPD/DS redirects bile to the distal ileum, where it is primarily reabsorbed and undergoes further microbial metabolism by the intestinal microbiota. The premature exposure of the distal intestine to bile induces an elevation in circulating bile acids and modulates lipid metabolism.

Ceramides, a class of specific bioactive lipids, are predominantly regarded as molecules that induce endoplasmic reticulum stress and trigger apoptosis in various cell types (11). Ceramide concentrations are strongly associated with the development of insulin resistance, obesity, diabetes, cardiovascular disease, and neurodegenerative diseases (12). Ceramides induce the activation of protein kinase C zeta, which subsequently inhibits Akt, a crucial mediator within the insulin signaling pathway in skeletal muscle and adipose tissue (13). In liver cells, ceramides disrupt the mitochondrial electron transport chain and inhibit fatty acid oxidation, and further impair insulin signaling (14). Considering that BPD/DS could improve insulin sensitivity and lipid metabolism in obesity patients, would the insulin resistance in PCOS patients be improved after BPD/DS? And do bile acids and ceramides involve in the mechanism of improvement? To the best of our knowledge, limited research has addressed these specific aspects. Intended to investigate the effects of BPD/DS on PCOS patients, we established models received BPD/DS.

## 2 Animals and methods

### 2.1 Animals

Twenty-five three-week-old female Sprague-Dawley rats (50-55g) (SLAC Experimental Animal Co., Ltd, Shanghai) were purchased for the establishment of PCOS models. The rats were housed individually under controlled environmental conditions (temperature: 20–26°C; relative humidity: 50%; 12-hour light-dark cycle) with ad libitum access to chow diet (60% fat, 5.24 kcal/g) and water, and bedding was changed daily. After 3-week acclimation, the PCOS model was generated via subcutaneous administration of dehydroepiandrosterone (DHEA; 6 mg per 100 g body weight dissolved in 0.2 mL tea oil) for 20 days (1). To evaluate the model’s validity, five rats were randomly selected and euthanized by intraperitoneal injection of pentobarbital sodium (100 mg per 1 kg body weight). Autopsy confirmed polycystic ovaries according to the AES diagnostic criteria (15). The remaining twenty PCOS rats were randomly allocated to two groups: the biliopancreatic diversion with duodenal switch (BPD/DS) group (n=10) and the Sham BPD/DS (S-BPD) group (n=10). DHEA was administered continuously to both groups of rats until euthanasia nine weeks postoperatively.

### 2.2 Surgical procedures

**Figure 1:**
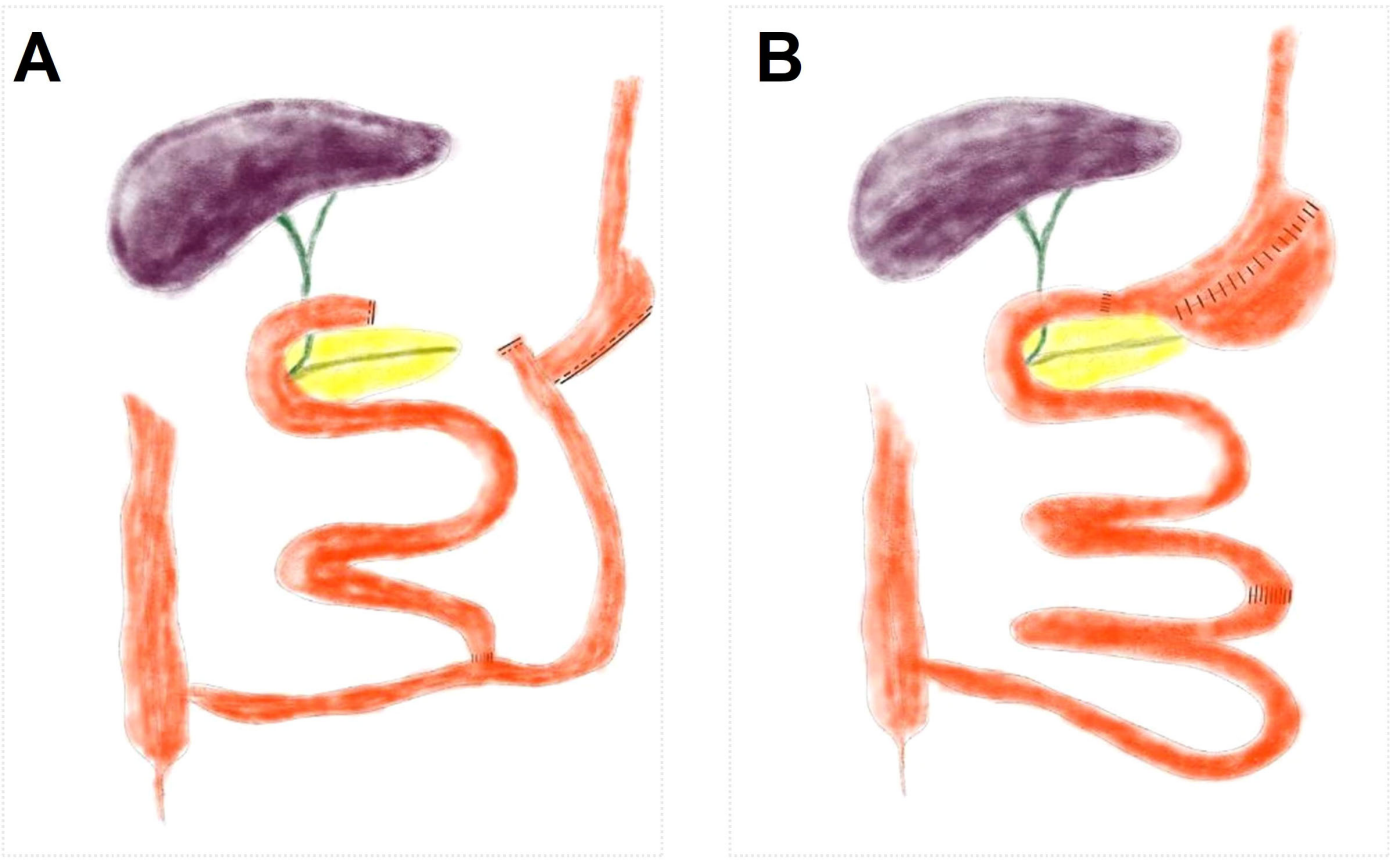
Schematic diagram of BPD/DS and S-BPD surgical procedure. **(A)** Sketch representation of BPD/DS surgical procedure. It included sleeve gastrectomy and transection of distal ileum. The distal ileum is anastomosed to the stomach, while the duodenum (or biliopancreatic limb) is anastomosed to the terminal ileum at 20 cm proximal to the ileocecal valve. **(B)** Sketch representation of S-BPD surgical procedure, which includes the same intestinal transection, but omits intestinal reconstruction. BPD/DS, the biliopancreatic diversion with duodenal switch group; S-BPD, the sham procedure of BPD/DS.

After rats fasted for 12 hours, BPD/DS and S-BPD surgical procedures were conducted within their respective surgical groups. Rats were anaesthetized by intraperitoneal injection of Ketamine (30 mg per 1 kg body weight) and pentobarbital sodium (40 mg per 1 kg body weight). As previously described (16), the BPD/DS included sleeve gastrectomy and gastrointestinal reconstructions (**Figure 1**). The distal ileum was transected 50 cm proximal to the ileocecal junction and subsequently anastomosed to the stomach. The proximal ileum, along with the duodenum containing bile and pancreatic juice, was anastomosed to the terminal ileum at 20 cm above the ileocecal junction. The S-BPD procedure involved the same intestinal transection, but did not undergo intestinal reconstruction. The intestine was anastomosed *in situ*, the other operating procedures were the same as those of BPD/DS. All rats were administered a non-residue diet (Ensure, Abbott, USA), comprising 15% fat, 56% carbohydrate, 26% protein, and 3% fiber and salt, which was maintained for a duration of 2 weeks. Rats were housed in individual cages routinely disinfected with 75% alcohol daily. Then, chow diet provided before surgery was given. The surgical procedures and protocols were approved and supervised by the institution ethics committee.

### 2.3 Bodyweight and estrous cyclicity

To minimize the influence of food intake, rats from both groups were pair-fed. Body weight was measured at 08:00 hours following an overnight fast. The estrous cycle was monitored via vaginal smears, based on cellular morphology and density, as previously described (17).

### 2.4 Insulin sensitivity tests

Oral glucose tolerance tests (OGTT) and insulin tolerance tests (ITT) were conducted preoperatively and at 4 and 8 weeks postoperatively (18). During OGTT, a dextrose solution (1 g per 1 kg body weight) was administered via intragastric gavage. Blood glucose levels from the tail vein were measured using an automated glucose meter at baseline and at 15, 30, 60, and 120 min post-administration. The area under the blood glucose curve (AUC_OGTT_) was calculated using the trapezoidal rule. During ITT, human insulin (0.75 mU per 1 kg body weight) was administered intraperitoneally. Blood glucose levels were also measured at baseline and at 15, 30, 60, and 120 min, and the area under the blood glucose curve (AUC_ITT_) was calculated.

### 2.5 Administration of bile acid sequestrant

**Figure 2:**
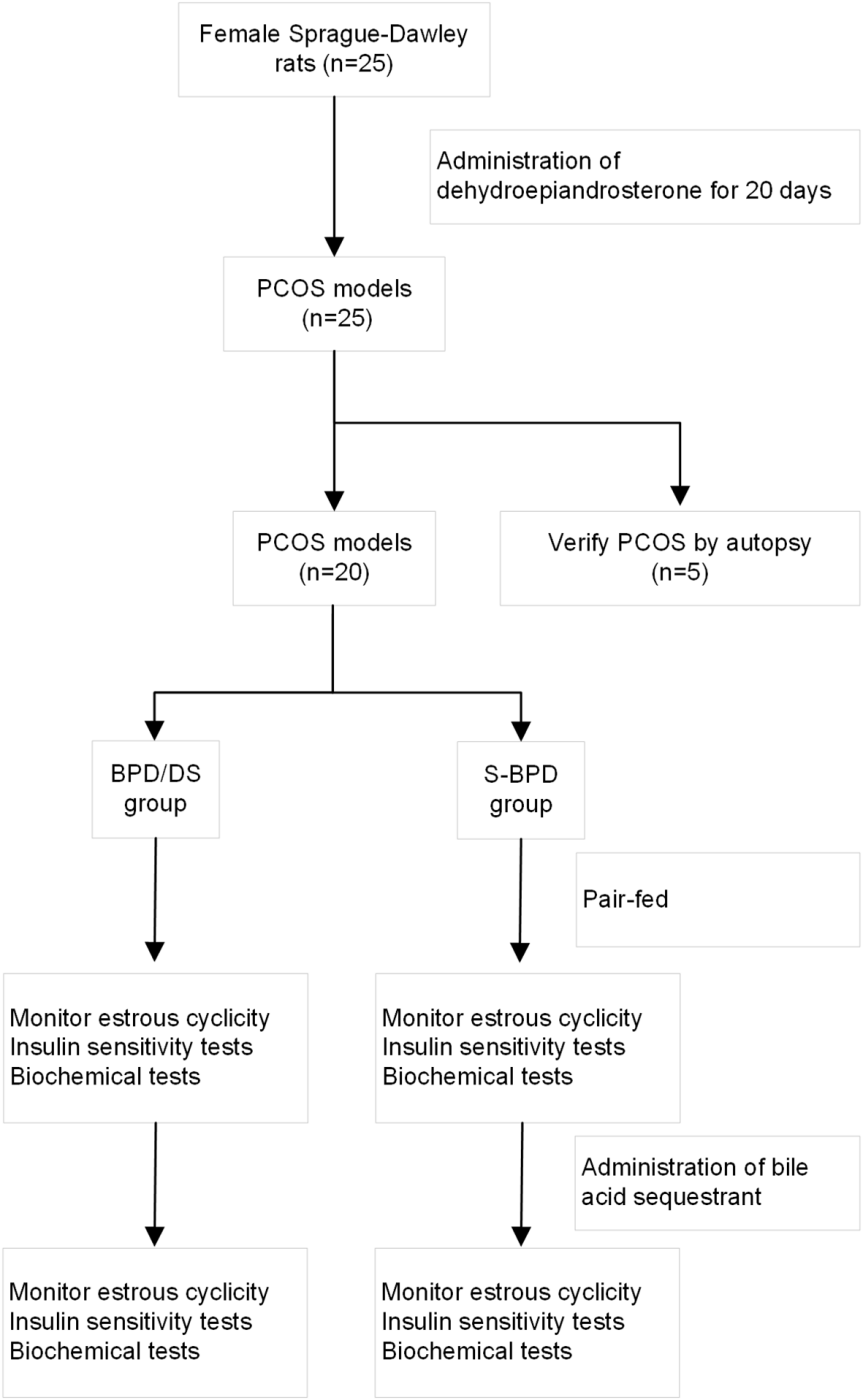
The study workflow. BPD/DS, the biliopancreatic diversion with duodenal switch group; S-BPD, the sham procedure of BPD/DS; PCOS, polycystic ovary syndrome.

During the ninth postoperative week, rats in the BPD/DS group were administered a chow diet containing 2% cholestyramine (CLS), a bile acid sequestrant that reduces the reabsorption of bile acids (19). To investigate changes in insulin sensitivity after bile acid chelation, oral glucose tolerance tests and insulin tolerance tests were also conducted. And the fasting blood samples were collected. All rats were euthanized 10 weeks after surgery by intraperitoneal injection of pentobarbital sodium (100 mg per 1 kg body weight). The workflow was showed in **Figure 2**.

### 2.6 Biochemical tests

Blood specimens were obtained via tail vein puncture in experimental rats. Subsequent to centrifugation at 3,000 × g for 15 minutes, serum samples underwent cryopreservation at −80°C pending biochemical analysis. The concentrations of estradiol (E2), progesterone, testosterone, anti-Müllerian hormone (AMH) and total bile acids were quantified using enzyme-linked immunosorbent assay kits sourced from R&D Systems (Minneapolis, MN, USA), in strict adherence to the manufacturer’s protocols. The concentrations of luteinizing hormone (LH), follicle-stimulating hormone (FSH), and insulin were assayed utilizing the Luminex 200 (Luminex Corp., Austin, TX, USA) in accordance with the manufacturer’s instructions.

Ceramides were extracted using the single-phase extraction method utilizing a butanol:methanol mixture (1:1 v/v) (20). The quantification of ceramides was conducted using liquid chromatography-mass spectrometry, Q-Exactive Orbitrap mass spectrometer (Thermo Fisher Scientific, Waltham, MA, USA) and high-performance liquid chromatography system Dionex Ultimate 3000 RS (Thermo Fisher Scientific) (21). C8 column Ascentis Express (2.7 μm, 2.1 × 100 mm) was bought from Merck Supelco. Briefly, the mobile phase composition for liquid chromatography consisted of mobile phase A (40% isopropanol, 8 mM ammonium formate and 2 mM formic acid) and mobile phase B (98% isopropanol, 8 mM ammonium formate and 2mM formic acid). The gradient used was as follows: 0-1.5 min, linear gradient from 0% to 20% B; to 28% B over 5.5min; to 35% B over 1 min, to 65% B over 16min, and to 100% B over 1min. The flow rate of the mobile phase was set at 0.2 mL/min, and the injection volume was 10µL. Mass spectrometer operated in full scan mode with positive and negative polarity switching at 70,000 resolution at 200 mass/charge ratio (*m/z*), the detection range was 140 to 1300 *m/z*. The spray voltage was set to 3.5 kV for positive mode and negative mode, and the capillary temperature and the probe heater temperature was maintained at 250 and 190°C, respectively. The sheathe and auxiliary gases were set to 34 and 13 units, respectively.

### 2.7 Statistical analysis

Quantitative data were presented as the mean ± standard deviation. Differences between two groups were analyzed using Student’s t-test with SPSS Statistics software (version 24.0, IBM, USA), and *P*-values<0.05 were considered statistically significant.

## 3 Results

### 3.1 Rat models

Rats were administered of DHEA to induce PCOS. After the 20-day subcutaneous injection, the estrous cycles of rats were thoroughly absent by vaginal smears detection. Rats showed glucose tolerance impairment and insulin resistance. And the five euthanized rats showed polycystic ovary structures. The rat models for PCOS were successfully established.

BPD/DS surgical procedures and sham procedures were carried out smoothly in the corresponding surgical groups. One BPD/DS model rat succumbed during the first postoperative week. Postmortem examination attributed the mortality to intestinal leakage and subsequent abdominal infection. The remaining rats remained free of severe complications.

### 3.2 Bodyweight and reproductive disorders improvement

**Figure 3:**
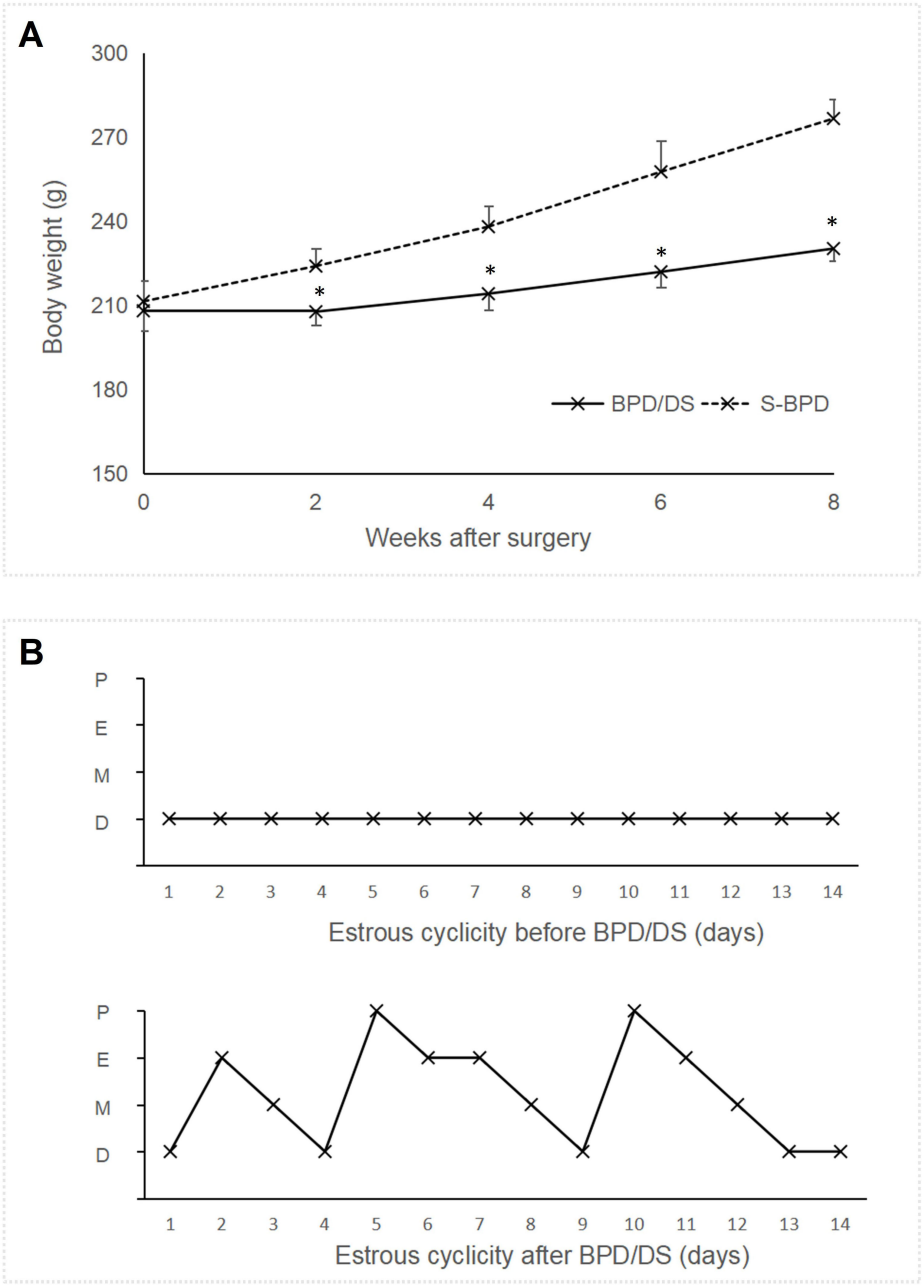
Body weight and menstrual cyclicity of two group rats. **(A)** Body weight of two groups. The body weight of the S-BPD group was significantly greater than that of the BPD/DS group after surgery (*P <*0.05). **(B)** Estrous cyclicity in the PCOS rats before surgery and after surgery. BPD/DS, biliopancreatic diversion with duodenal switch group; S-BPD, the sham procedure of BPD/DS; D, diestrus; E, estrus; M, metestrus; P, proestrus; PCOS, polycystic ovary syndrome. * there is significant difference between the two groups.

No significant difference was observed in body weight between the BPD/DS group and the S-BPD group prior to surgery (208.0 ± 6.9 g vs 211.4 ± 7.4 g, *P* =0.311, **Figure 3**). Both group were pair-fed, the body weight of the S-BPD group rats was 276.5 ± 7.0 g, while that of the BPD/DS group rats was 228.9 ± 4.2 g, which was significantly less than the S-BPD group at 8 weeks post-surgery (*P <*0.001).

Both groups exhibited acyclicity prior to surgery due to PCOS. The BPD/DS group initiated restoration of normal cyclicity commencing at 4 weeks post-surgery, with cyclicity nearly fully recovered by the 8^th^ postoperative week (**Figure 3**).

### 3.3 BPD/DS improve insulin sensitivity

**Figure 4:**
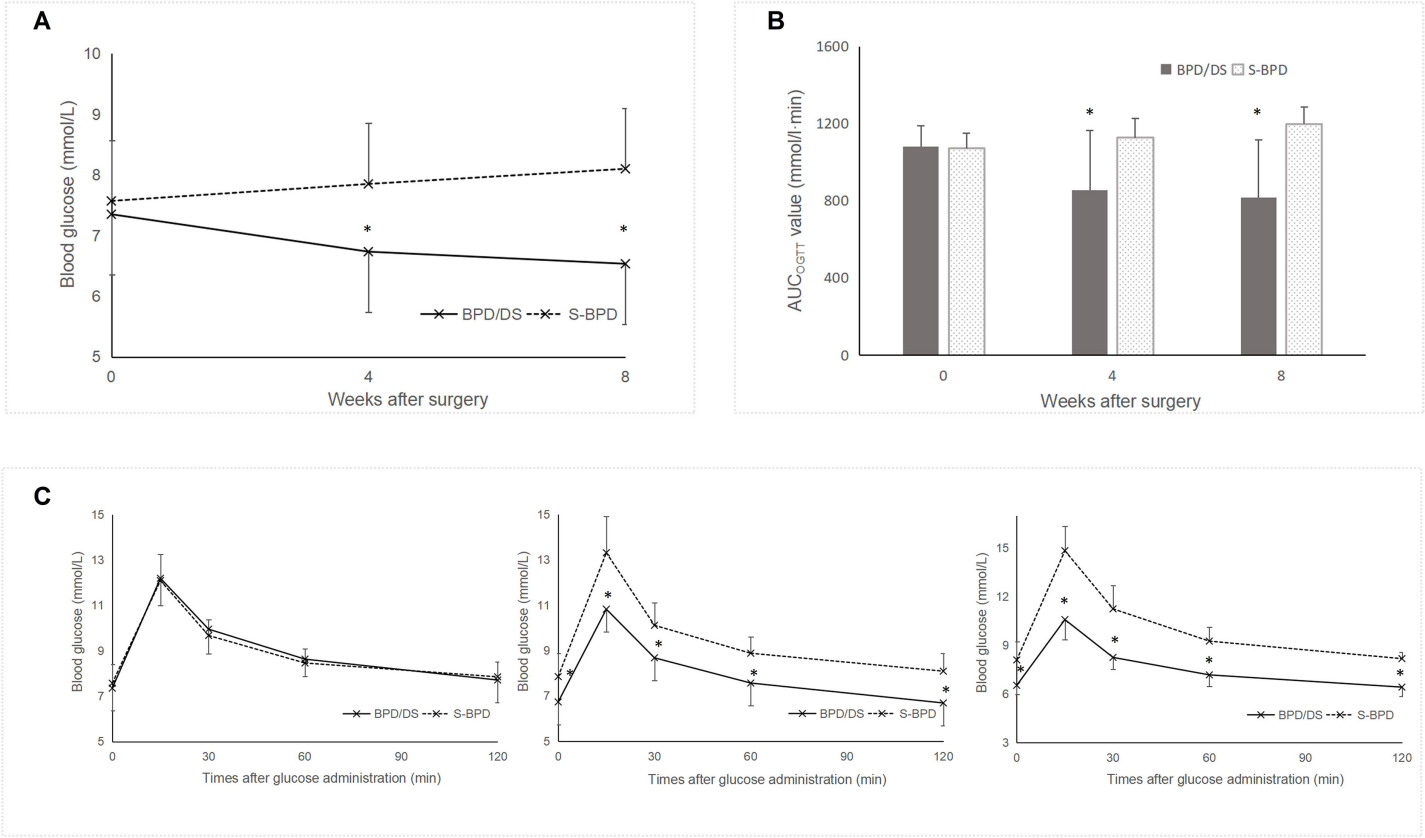
Glucose metabolism profiles in the two groups. **(A)** Fasting glucose levels during the perioperative period. Lower fasting glucose level was observed in the BPD/DS group postoperatively. **(B)** AUC_OGTT_ value during the perioperative period. The AUC_OGTT_ value was significantly higher in the S-BPD group than in the BPD/DS group postoperatively (*P <*0.05). **(C)** Glucose curves obtained during the OGTT performed preoperatively, at postoperative week 4 and at postoperative week 8. AUC, area under curve; OGTT, oral glucose tolerance test; BPD/DS, biliopancreatic diversion with duodenal switch group; S-BPD, the sham procedure of BPD/DS. * there is significant difference between the two groups.

**Figure 5:**
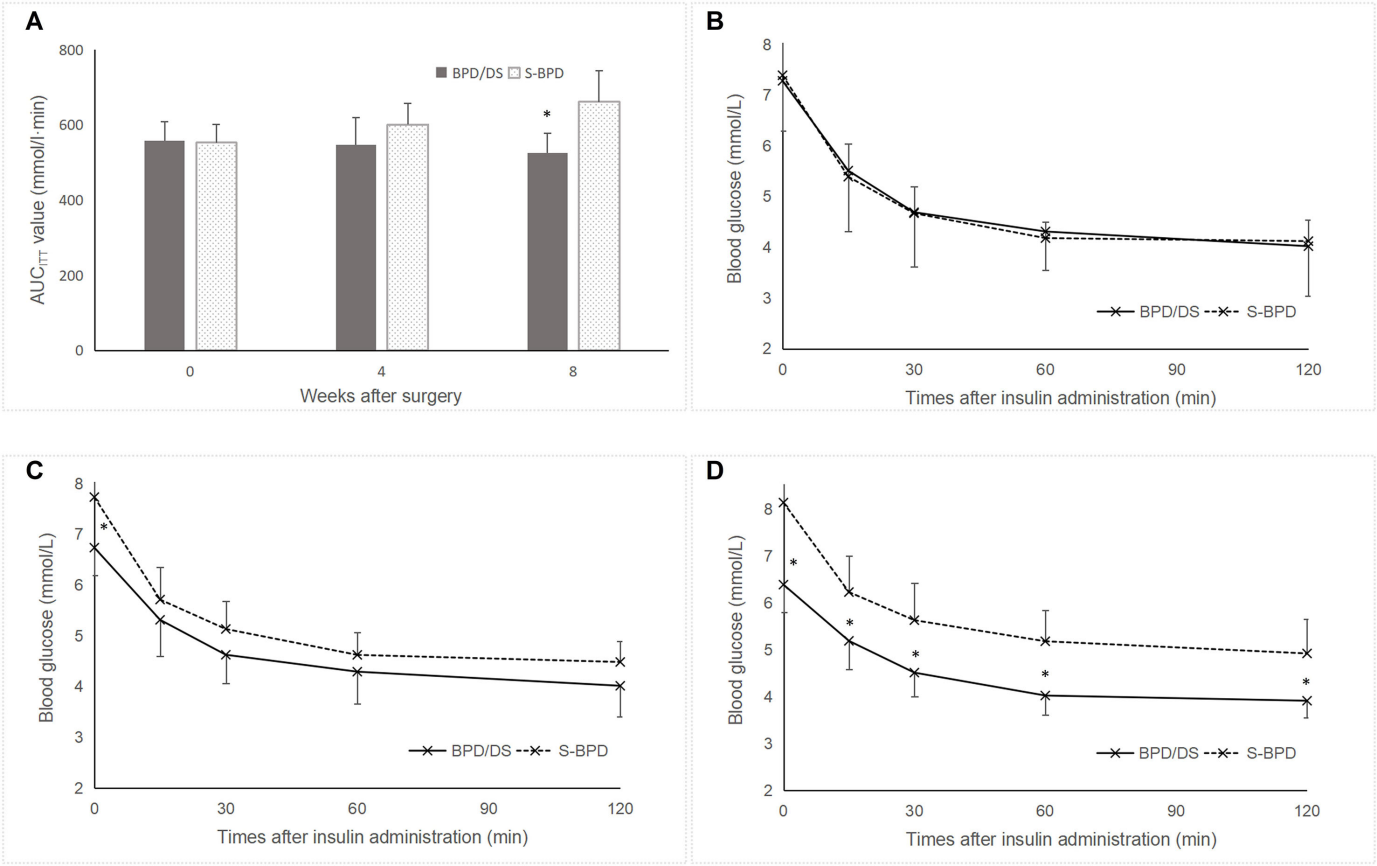
Insulin resistance in the two groups. **(A)** AUC_ITT_ values during the perioperative period. The S-BPD group exhibited significantly higher AUC_ITT_ value compared to the BPD/DS group at postoperative week 8 (*P <*0.05). **(B)** Glucose curve during the ITT prior to surgery. **(C)** Glucose curve during the ITT at postoperative week 4. **(D)** Glucose curve during the ITT at postoperative week 8. AUC, area under curve; ITT, insulin tolerance test; BPD/DS, biliopancreatic diversion with duodenal switch group; S-BPD, the sham procedure of BPD/DS. * there is significant difference between the two groups.

Postoperatively, glucose tolerance and insulin sensitivity exhibited significant improvement in the BPD/DS group relative to the S-BPD group. Blood glucose levels in the BPD/DS rats decreased from 7.4 ± 1.0 mmol/L preoperatively to 6.5 ± 0.5 mmol/L by postoperative week 8 (*P* =0.041, **Figure 4**). The AUC_OGTT_ value exhibited a significant reduction from 1081.5 ± 108.7 mmol/L·min to 818.3 ± 297.3 mmol/L·min (*P* =0.001, **Figure 4**). Additionally, the AUC_ITT_ value decreased from 557.3 ± 50.7 mmol/L·min to 525.6 ± 52.3 mmol/L·min (*P* =0.037, **Figure 5**). The OGTT and ITT blood glucose curves preoperatively and postoperatively are presented in **Figures 4** and **5**. Furthermore, the S-BPD group exhibited an increase in blood glucose level from 7.6 ± 0.8 mmol/L to 8.1 ± 1.1 mmol/L at postoperative week 8 (*P* =0.008). Correspondingly, the AUC_OGTT_ value increased from 1071.9 ± 78.2 mmol/L·min to 1198.6 ± 86.4 mmol/L·min (*P* =0.001), while the AUC_ITT_ value increased from 553.1 ± 48.2 mmol/L·min to 661.9 ± 81.8 mmol/L·min (*P* =0.037).

Preoperatively, no significant difference was observed between the two groups regarding the fasting blood glucose level (7.4 ± 1.0 mmol/L versus 7.6 ± 0.8 mmol/L, *P* =0.594), the AUC_OGTT_ value (1081.5 ± 108.7 mmol/L·min versus 1071.9 ± 78.2 mmol/L·min, *P* =0.823) or the AUC_ITT_ value (557.3 ± 50.7 mmol/L·min versus 553.1 ± 48.2 mmol/L·min, *P* =0.849). Postoperatively, fasting blood glucose levels were significantly lower in the SPB group compared to the S-BPD group (6.5 ± 0.5 mmol/L versus 8.1 ± 1.1 mmol/L, *P <*0.001). Similarly, AUC_OGTT_ values were significantly reduced in the SPB group relative to the S-BPD group (818.3 ± 297.3 mmol/L·min versus 1198.6 ± 86.4 mmol/L·min, *P <*0.001). In addition, AUC_ITT_ values were significantly lower than those in the S-BPD group (525.6 ± 52.3 mmol/L·min versus 661.9 ± 81.8 mmol/L·min, *P* =0.001).

### 3.4 Hormone level changes after BPD/DS

**Figure 6:**
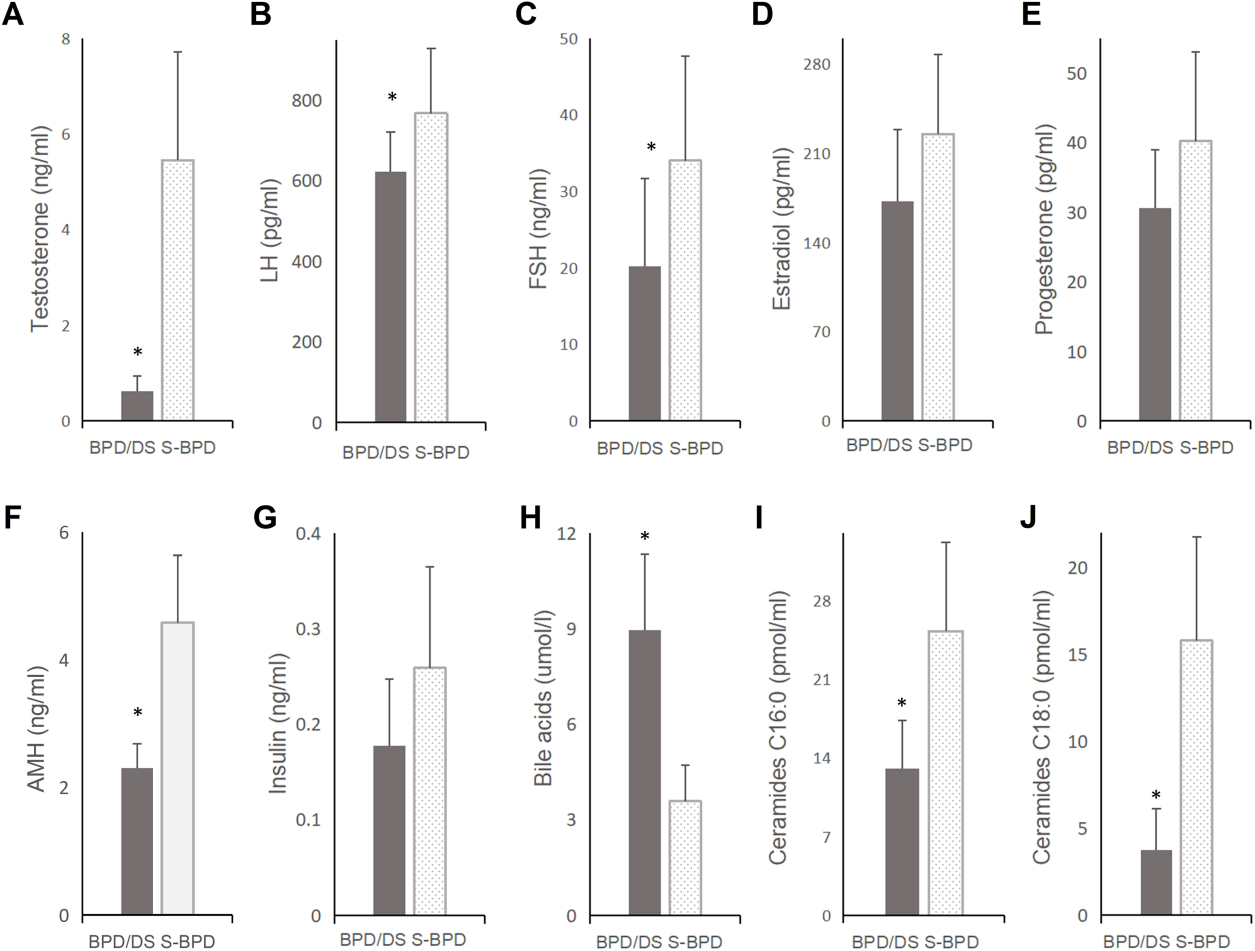
Serum hormones, bile acids and ceramides concentrations in PCOS rats following BPD/DS treatment. **(A)** Testosterone concentrations, **(B)** LH concentrations, **(C)** FSH concentrations, **(D)** estradiol concentrations, **(E)** progesterone concentrations, **(F)** AMH concentrations, **(G)** insulin concentrations, **(H)** total bile acids concentrations, **(I)** ceramides C_16:0_ concentrations and **(J)** ceramides C_18:0_ concentrations. AMH, anti-Müllerian hormone; FSH, follicle-stimulating hormone; LH, luteinizing hormone. BPD/DS, biliopancreatic diversion with duodenal switch group; S-BPD, the sham procedure of BPD/DS group. * there is significant difference between the two groups.

At the eighth postoperative week, the concentrations of serum testosterone, LH, FSH and AMH of the BPD/DS group were significantly lower than those of the S-BPD group (**Figure 6**). The testosterone level was 0.62 ± 0.32 ng/mL in the BPD/DS group, while 5.46 ± 2.27 ng/mL in the S-BPD group (*P* =0.001). The LH level was 623.11 ± 99.18 pg/mL in the BPD/DS group, while 767.90 ± 161.32 ng/mL in the S-BPD group (*P* =0.033). The FSH level was 20.23 ± 11.48 ng/mL in the BPD/DS group, while 34.04 ± 131.69 ng/mL in the S-BPD group (*P* =0.030). The AMH level was 2.30 ± 0.38 ng/mL in the BPD/DS group, while 4.58 ± 1.05 ng/mL in the S-BPD group (*P <*0.001).

No statistically significant differences were observed in E2, progesterone, or insulin levels between the two groups (**Figure 6**). The E2 level was 172.67 ± 55.71 pg/mL in the BPD/DS group, compared to 225.30 ± 61.99 pg/mL in the S-BPD group (*P* =0.069). The progesterone level was 30.66 ± 8.37 pg/mL in the BPD/DS group, compared to 40.24 ± 12.89 pg/mL in the S-BPD group (*P* =0.075). The insulin level was 0.18 ± 0.07 ng/mL in the BPD/DS group, compared to 0.26 ± 0.11 pg/mL in the S-BPD group (*P* =0.072).

### 3.5 Ceramides and bile acids level changes

Elevated bile acid levels and reduced ceramide levels were observed in the BPD/DS group. The total bile acids concentration was 8.97 ± 2.39 μmol/L in the BPD/DS group, significantly higher than that of the S-BPD group (3.59 ± 1.14 μmol/L, *P <*0.001, **Figure 6**).

The serum C_16:0_ ceramide level in the BPD/DS group was 13.11 ± 4.28 pmol/mL, significantly lower than that of the S-BPD group (25.30 ± 7.92 pmol/mL, *P <*0.001, **Figure 6**). Similarly, the serum C_18:0_ ceramide level measured 3.73 ± 2.38 pmol/mL in the BPD/DS group, which was also significantly lower than that of the S-BPD group (15.80 ± 5.98 pmol/mL, *P <*0.001).

### 3.6 Cholestyramine deteriorates insulin sensitivity

**Figure 7:**
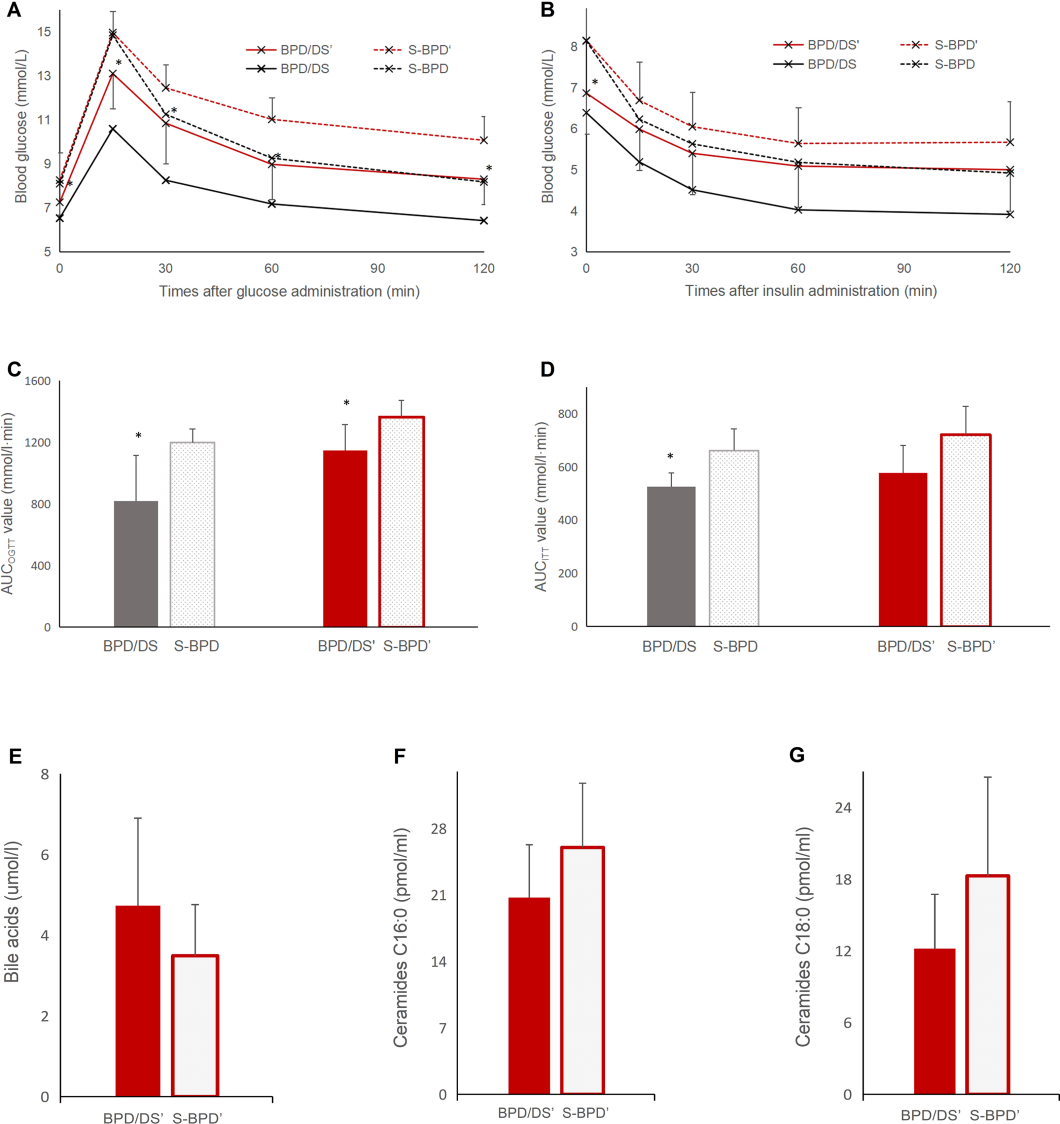
Glucose profiles during OGTT and ITT after bile acid sequestrant administration. **(A)** Glucose curve during OGTT after bile acid sequestrant administration. **(B)** Glucose curve during ITT after bile acid sequestrant administration. **(C)** AUC_OGTT_ value before and after bile acid sequestrant administration. **(D)** AUC_ITT_ value before and after bile acid sequestrant administration. **(E)** total bile acids concentrations, **(F)** ceramides C_16:0_ concentrations and **(G)** ceramides C_18:0_ concentrations after bile acid sequestrant administration. OGTT, oral glucose tolerance test; ITT, insulin tolerance test; BPD/DS, biliopancreatic diversion with duodenal switch group; BPD/DS’, biliopancreatic diversion with duodenal switch group with bile acid sequestrant administration; S-BPD, the sham procedure of BPD/DS group; S-BPD’, the sham procedure of BPD/DS group with bile acid sequestrant administration. * there is significant difference between the two groups.

At the ninth postoperative week, the cholestyramine, bile acid sequestrant, was given to the BPD/DS group rats for 1 week. And blood glucose levels in the BPD/DS group increased from 6.5 ± 0.5 mmol/L to 7.2 ± 0.6 mmol/L (*P* =0.032), yet remained significantly lower than those observed in the S-BPD group (8.3 ± 1.3 mmol/L, *P* =0.043, **Figure 7**). During OGTT, the AUC_OGTT_ value in the BPD/DS group increased from 818.3 ± 297.3 mmol/L·min to 1147.9 ± 167.9 mmol/L·min (*P* =0.007), but was also significantly lower than that of the S-BPD group (1364.5 ± 109.0 mmol/L·min, *P* =0.004). During the ITT, the AUC_ITT_ value in the BPD/DS was also significantly increased from 525.6 ± 52.3 mmol/L·min to 577.7 ± 102.9 mmol/L·min (*P* =0.023, **Figure 7**), and was similar to that of the S-BPD group (721.5 ± 106.2 mmol/L·min, *P* =0.116).

### 3.7 Cholestyramine attenuates intestinal bile acid absorption

After bile acid chelation, the level of bile acid was 4.73 ± 2.17 μmol/L in the BPD/DS group, less than that before chelation (8.97 ± 2.39 μmol/L, *P <*0.001), and similar with that of the S-BPD group (3.49 ± 1.27 μmol/L, *P* =0.14, **Figure 7**).

The serum C_16:0_ ceramide level was 20.72 ± 5.57 pmol/mL in the BPD/DS group, higher than that before chelation (13.11 ± 4.28 pmol/L, *P* =0.010), and similar with that of the S-BPD group (26.00 ± 6.77 pmol/mL, *P* =0.083, **Figure 7**). The serum C_18:0_ ceramide level was 12.18 ± 4.55 pmol/mL in the BPD/DS group, higher than that before chelation (3.73 ± 2.38 pmol/L, *P* =0.001), and similar with that of the S-BPD group (18.27 ± 8.26 pmol/mL, *P* =0.067).

## 4 Discussion

PCOS is considered to be a heterogeneous disease, characterized by anovulation, androgen excess and metabolic syndrome. Insulin resistance and obesity are tightly correlated with PCOS. Within the ovaries, insulin functions synergistically with LH to stimulate androgen synthesis. High level of insulin resulted from insulin resistance led to high level of testosterone and other androgens (22). Furthermore, elevated insulin levels inhibit hepatic synthesis of sex hormone-binding globulin, consequently elevating testosterone levels, and inducing follicular stoppage and ovulatory failure (23).

Multiple bariatric procedures, including sleeve gastrectomy, Roux-en-Y gastric bypass, and adjustable gastric banding, have been reported to improve reproductive outcomes in previously infertile women (24). These surgical interventions enhance insulin sensitivity and normalize reproductive hormone levels, thereby facilitating ovulatory function. In the present study, we similarly observed significant improvements in reproductive disorders and insulin sensitivity following BPD/DS. The BPD/DS group commenced recovery of normal estrous cyclicity at 4 weeks post-surgery, with cyclicity largely restored by the eighth postoperative week. Serum testosterone, FSH, and LH concentrations were significantly decreased in the BPD/DS group. Moreover, fasting glucose levels, AUC_OGTT_ values and AUC_ITT_ values were significantly reduced. These metabolic improvements occurred independently of weight loss. Notably, insulin sensitivity demonstrated clinical improvement within the initial postoperative weeks, even in the absence of significant weight reduction.

Unger et al. previously described “lipotoxicity” that the lipids accumulation caused varied organ dysfunction underlied obesity-related diseases (25). Ceramide were recognized as “the most damaging lipid”, because they could induce apoptosis of β-cells (26), also impair insulin signaling pathways and reduce glucose uptake in 3T3L1 adipocytes (27). Pharmacological inhibition or genetic ablation of key enzymes critical for ceramide biosynthesis ameliorates diabetic phenotypes. For instance, administration of the serine palmitoyltransferase (SPT) inhibitor myriocin to Zucker diabetic fatty rats prevents hyperglycemia onset, and enhances insulin sensitivity (28).

Ceramides are essential precursors of most of the complex sphingolipids, which performs structural roles in cell membranes. When caloric intake exceeds metabolic demand, ceramides act as intracellular signaling molecules indicative of free fatty acid abundance, initiating adaptive responses that enable cellular adaptation to lipid overload (12). Ceramides serve as a biomarkers for cardiovascular disease and diabetes mellitus in humans independent of cholesterol (29). Ceramide synthases catalyze the acylation of the sphingoid backbone using specific fatty acyl-CoAs, generally ranging from 14 to 26 carbon atoms. The biological impact of ceramides is contingent upon the chain length and saturation level of their constituent fatty acyl moieties. Ceramide possessing a 16-carbon acyl chain (C_16:0_ ceramide) has been certified to implicate in obesity and insulin resistance (30). Similarly, the accumulation of C_18:0_ ceramides induces insulin resistance through inhibition of AKT activity, impairing mitochondrial respiration and increasing reactive oxygen species production (31). The degree of insulin resistance exhibits a positive correlation with serum levels of both C_16:0_ and C_18:0_ ceramides (30). Elevated serum concentrations of C_16:0_ and C_18:0_ ceramides are positively associated with the incidence of T2DM and inversely associated with pancreatic β-cell function (32).

Although no significant difference in insulin levels was observed between the two groups, the BPD/DS group exhibited lower serum concentrations of C_16:0_ and C_18:0_ compared to the S-BPD group. Accumulating evidence indicates that circulating ceramide concentrations are intricately linked to lipid regulation, as circulating lipids serve as the primary substrate reservoir for ceramide synthesis (33). Lipids preferentially utilize an evolutionarily conserved pathway for trafficking to the endoplasmic reticulum (ER), where they undergo assembly or esterification into neutral lipids or serve as precursors for generating metabolic intermediates and signaling molecules (34). The sequestration of fatty acids into neutral lipids, such as triglycerides within lipid droplets (LDs), prevents ceramide accumulation by reducing the availability of free fatty acid substrates for ceramide synthesis at the ER (35). Disruption of LD biogenesis, exemplified by adipose-specific knockout of SEIPIN, induces a significant increase in C_18:0_ ceramides (36). After biliopancreatic diversion, the level of bile acids increased significantly. High level of bile acids enhance lipid metabolism. When the bile acid sequestrant was given, the level of bile acids decreased. Interestingly, levels of C_16:0_ and C_18:0_ ceramides rose significantly, and similar with that of the S-BPD group. Furthermore, insulin resistance of PCOS was also deteriorated. Consequently, bile acids and ceramides contribute to the improvement of PCOS after BPD/DS.

Several limitations exist in our investigations. A significant limitation is the incomplete investigation of the ceramide profile. Future research will focus on the signaling pathways downstream of ceramides. In this study, preliminary results confirmed the improvement of insulin resistance in PCOS rats following BPD/DS, with bile acids and ceramides implicated in this enhancement. A comprehensive understanding of the mechanisms underlying the improvement in insulin sensitivity after BPD/DS may facilitate the development of minimally invasive therapeutic strategies for PCOS and T2DM. Clinical investigations into ceramide-based therapies should be focused in future.

## 5 Conclusions

Our study results demonstrate that BPD/DS improved insulin resistance in PCOS models, concomitant with a reduction in serum ceramide concentrations. Administration of a bile acid sequestrant reversed this improvement in insulin resistance, an effect paralleled by elevated ceramide levels.

## Data Availability

The datasets presented in this study can be found in online repositories. The names of the repository/repositories and accession number(s) can be found in the article/supplementary material.
